# Standardization and Control of Grignard Reactions in a Universal Chemical Synthesis Machine using online NMR

**DOI:** 10.1002/anie.202106323

**Published:** 2021-08-06

**Authors:** Martin Bornemann‐Pfeiffer, Jakob Wolf, Klas Meyer, Simon Kern, Davide Angelone, Artem Leonov, Leroy Cronin, Franziska Emmerling

**Affiliations:** ^1^ Department 1: Analytical Chemistry, Reference Materials Bundesanstalt für Materialforschung und -prüfung Richard-Willstätter-Straße 11 12489 Berlin Germany; ^2^ Chair of Chemical and Process Engineering Technische Universität Berlin Marchstr. 23 10587 Berlin Germany; ^3^ S-PACT GmbH Burtscheiderstr. 1 52064 Aachen Germany; ^4^ School of Chemistry University of Glasgow Glasgow G12 8QQ UK

**Keywords:** Grignard reaction, NMR spectroscopy, process analytical technology, process control

## Abstract

A big problem with the chemistry literature is that it is not standardized with respect to precise operational parameters, and real time corrections are hard to make without expert knowledge. This lack of context means difficult reproducibility because many steps are ambiguous, and hence depend on tacit knowledge. Here we present the integration of online NMR into an automated chemical synthesis machine (CSM aka. “Chemputer” which is capable of small‐molecule synthesis using a universal programming language) to allow automated analysis and adjustment of reactions on the fly. The system was validated and benchmarked by using Grignard reactions which were chosen due to their importance in synthesis. The system was monitored in real time using online‐NMR, and spectra were measured continuously during the reactions. This shows that the synthesis being done in the Chemputer can be dynamically controlled in response to feedback optimizing the reaction conditions according to the user requirements.

## Graphical abstract

A universal chemical synthesis platform was extended by online NMR to allow adjustments of the encoded reactions on the fly. The approach was demonstrated on the class of Grignard reactions showing robust analytical results under harsh conditions. Real‐time process analytics and the use of straightforward feedback control algorithm enhance the usability of available synthesis formula considering existing deviations.

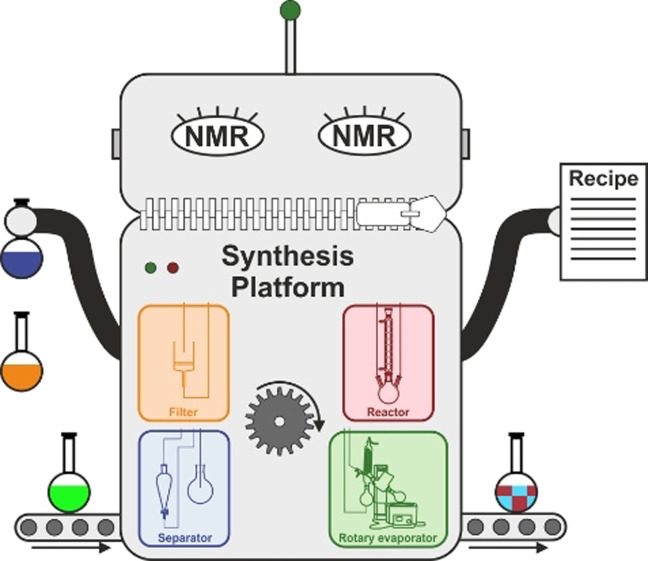

In the pharmaceutical and chemicals industry automation is well established for the execution of routine tasks, but these are often limited to well‐defined processes that are narrow in scope. Similarly, in the chemistry laboratory bench scale synthesis automation is still in its infancy due to the cost of the hardware, and lack of universal software standards. Recently, a new paradigm for digital chemistry, using an universal abstraction of chemical synthesis was developed.^[1]^ This means that almost all chemical processes can now not only be automated, but universally programmed with a simple method that does not require complex programming. As such the reliable automatic execution of classical production processes require automatically positioning of tools on lines using sensor feedback, and similarly automated chemical transformations require information about the chemical state of a reaction. This information could be used to control the reaction “on the fly” or real‐time via the automatic processing of the analytical data. This is undeniably more challenging than positioning but would help to reduce reaction time, impurities, and reagent consumption. Also, automation serves as an enabling tool making the synthesis of new molecules accessible to non‐chemists, as shown for oligopeptide,^[2]^ ‐nucleotide^[3]^ and glycan^[4]^ synthesis. A biologist can synthesize an oligopeptide by desired sequence, without knowledge of protecting group strategies for example. Recent advances in automated flow synthesis of peptides allow fast^[5]^and reliable synthesis of long protein chains up to 164 amino acids.^[6]^ Aiming at a broader scope of chemistry and frameworks, automated batch and flow synthesis platforms, suitable for performing different chemical reactions with one platform were established in the past years.^[7]^ Whereas all platforms target the reduction of manual hands‐on work during the chemical synthesis, other objectives may differ. Extensive and recent overviews are given by Trobe and Burke^[8]^ and Wang et al.^[9]^ Currently, research focusses on automation of synthesis route planning and development,^[10]^ the automatic execution of chemical synthesis literature,^[1]^ and the use of machine learning accelerating experimental data gathering.^[11]^

The goal of automation is not just saving time, but the digitization of chemistry. The latter is not just accomplished by adding control systems, but by developing a digital abstraction and hard link by which conditions and work steps are versioned, and linked to verified results. This approach should be able to improve the quality of chemistry and reaction databases with contextual data well suited for subsequent reuse, for example, for machine learning.^[12]^ On‐line or in‐line optical/vibrational spectroscopy like UV/Vis, IR, Raman are well established in industry and some automated platforms.[^7c^, ^7d^, ^13^] Due to recent developments benchtop NMR instruments are becoming increasingly attractive for Process Analytical Technology (PAT).^[14]^ NMR spectroscopy in combination with robust data evaluation offers reliable and easily accessible relative quantitative results without prior calibration and was therefore chosen as analytical method. Further, application of benchtop NMR in combination with automated synthesis platforms was reported just in a few publications,[^13^, ^14d^, ^15^] so far.

The “Chemputer”[^1^, ^16^] is the first universal automated synthesis platform that was designed to implement the abstraction of chemical reactions in a universal manner according to their unit operations: additions, transfers, or physical manipulations (heating, stirring) performed in modules, for example, a flask with a condenser. These unit operations can be programmed, and the modularity of code and hardware allows application to a broad range of reactions. Thus far, the Chemputer relies upon precise instructions, yet without PAT and processing of chemical information, can only follow well‐defined synthesis routes programmed with fixed quantities and reaction times. So far, analysis was performed on reaction products, which is sufficient for optimized routes. Integration of PAT might entail significant cost, but also be helpful in cases like i) heterogeneous reactions with varying time scales or reactions with initiation times, ii) thermal runaway reactions, iii) unknown quantitative information (concentration, purity, amount) on starting materials including multi‐stage synthesis with required adaption of subsequent stages, iv) reaction optimization and v) avoiding side‐reactions/overreaction. In the cases mentioned, online analysis offers insights into reaction progress, present species, and concentrations, reveals optimization potential, and allows for faster malfunction identification. Feedback control will in turn lead to increased robustness, decreased chemical waste generation and energy consumption by reducing waiting times.

Herein we show the successful implementation of such feedback control for different starting materials in the Grignard reaction, selected to be the first benchmark candidate to validate the concept of building a self‐optimizing Chemputer. Due to harsh measurement conditions caused by evolution of solids, the reliable use of online NMR measurement is one key aspect which will be addressed in the research presented here.

The feasibility of the universal synthetic and analytical approach presented was demonstrated for a prototypical reaction class, the Grignard reaction (see Figure 1). This reaction class was chosen because: (1) Grignard couplings are applied in synthetic routes to pharmaceuticals and fine chemicals; (2) The initiation time of the heterogeneous Grignard formation may differ due to varying activation states of the magnesium involved: (3) The preparation of Grignard reagents are highly exothermic reactions. Due to the involvement of solid species in the chemical reaction, sampling and chemical analysis with NMR spectroscopy or any other method is challenging. As the reaction is highly exothermic, reaction monitoring is mandatory to shorten reaction times while ensuring safety. The evolution of Grignard reagents was already monitored via NMR^[17]^ but so far, the coupling of Grignard reagents to an aromatic aldehyde was not investigated using online methods. Further experimental details of the procedure are given in the Supporting Information.

**Figure 1:**
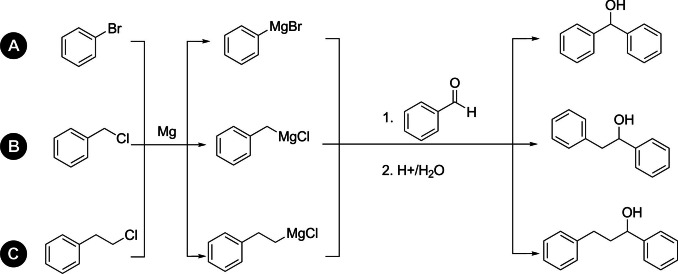
Examined reactions including Grignard reagent formation. A: Synthesis of Diphenylmethanol; B: Synthesis of 1,2‐Diphenylethanol; C: Synthesis of 1,3‐Diphenylpropan‐1‐ol.

The Chemputer employed in this study was developed by the Cronin Group, University of Glasgow^[16a]^ and can transfer liquids between modules with syringe pumps and six‐way selection valves actuated by the control software. Depending on synthetic needs, different modules can be combined, in this investigation that was a tempered reactor, liquid‐liquid separator, and rotary evaporator. The original platform can be extended by analytical devices, in our case a compact NMR instrument. In Figure 2, the design of the Chemputer and the herein developed analytical module including the primary communication infrastructure are shown.

**Figure 2:**
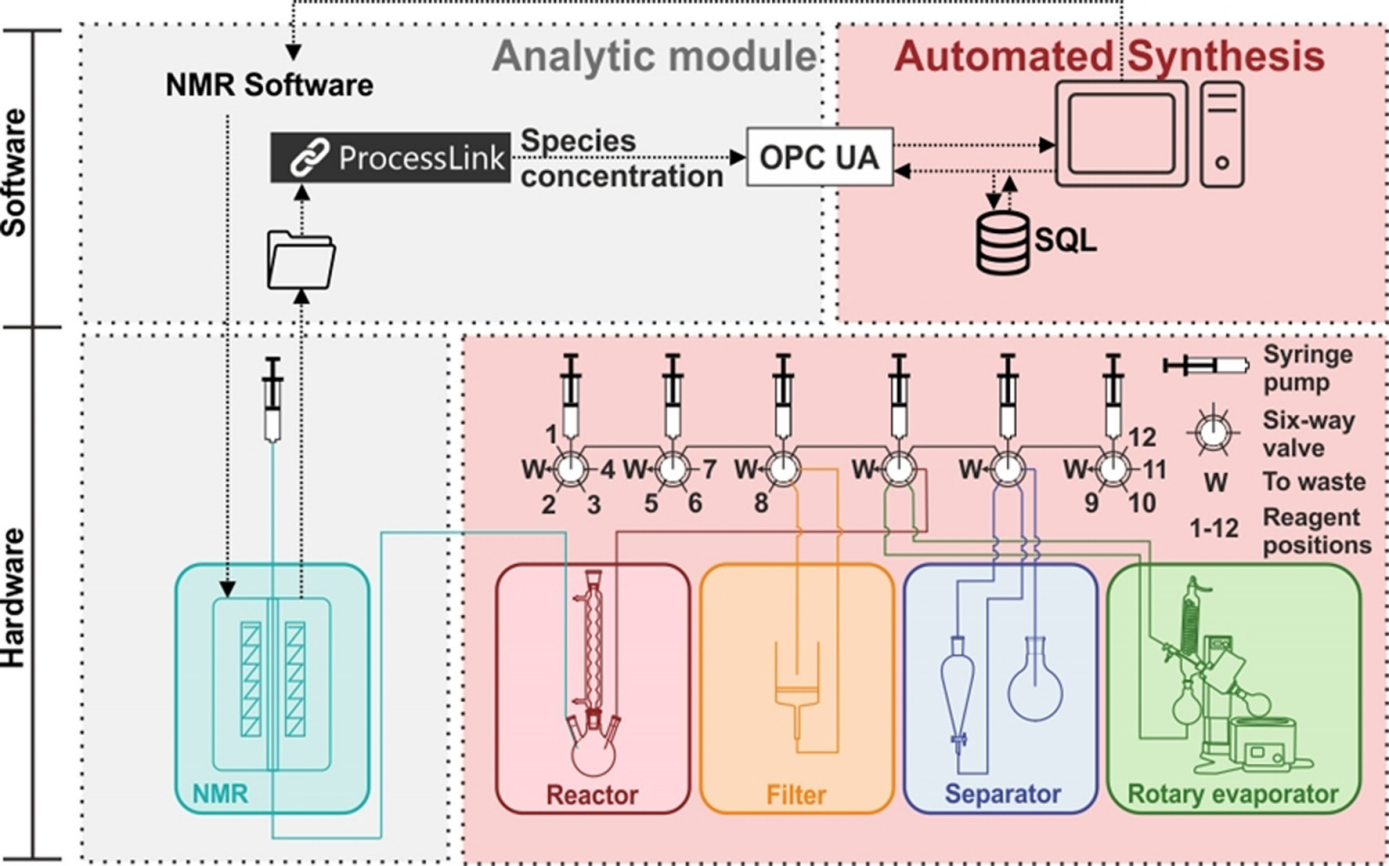
Schematic representation of the Chemputer and the analytical module with a Benchtop NMR. Upper part: Software implementation of the analytical module within the automated synthesis platform (Chemputer by Cronin et al.^[16a]^) and present interfaces including an Open Platform Communications Unified Architecture (OPC UA) interface and a Structured Query Language (SQL) database; lower part: the automated synthesis platform hardware with the newly added analytical module.

Interchangeability of the analytical method and evaluation software is ensured through a SQL database for information transfer, only requiring a small interface for for example, OPC UA based communication between SQL and analytical software. The underlying concept is the separation of the Chemputer and its respective controlling software (the Chempiler and analytical labware python modules)^[15]^ as an executing platform with the ability to trigger analytical measurements from the processing of analytical data. The measurement results can be evaluated by any suitable algorithm implementation or human input and the resulting information, for example, species concentration or the binary information about the presence of a distinct spectral feature are fed back into the database. Decisions taken by the Chemputer are solely based on traceable values in the database, thereby achieving high transparency, modularity, and simple integration. The application is demonstrated by incorporation of compact NMR and PT100 thermocouple, evaluated by a custom and a commercial algorithm. This could be extended to simple pH measurements and basically any standard analytical method. NMR spectroscopy was chosen as applied PAT due to its matrix‐independent linearity between measured signal and species concentrations. This is especially advantageous when several reaction species varying in concentration are present, causing time‐consuming calibration effort for all established PAT (e.g. UV/Vis, Raman, and NIR), except for NMR, to obtain quantitative results. Compact NMR instruments benefit from lower cost, no need for cooling agents and portability compared to common NMR instruments but suffer from peak broadening and hence potential overlapping of single peaks due to lower field strengths. Several methods exist which tackle this common problem in (not only NMR) spectra evaluation, for example, CRAFT algorithm,^[18]^ quantum mechanical approaches,^[19]^ machine learning^[20]^ or indirect hard modeling (IHM).^[21]^ As IHM has proven itself to be a robust method,^[22]^ it was applied in this work. The method is described in detail by Marquardt et al.^[23]^

Prior to implementation of the control system, automated reactions with the NMR instrument incorporated were performed to investigate simple and efficient approaches for decision making. The peak areas are calculated for every species through IHM. Normalization through the number of protons per species results in the determination of the relative concentrations. These are dimensionless and the calibration‐free approach is sufficient in our case, but absolute concentrations are easily accessible by single‐point calibration.^[24]^ The reaction progress can be evaluated by calculation of two basic parameters: the concentration gradient of one species and the ratio of selected species concentrations (for further details, see Supporting Information).

The comparison of these parameters with a set threshold value allows for the decision whether the current step should be resumed or stopped. The applied feedback‐controlled decisions include:

1. The initiation of the organomagnesium halide formation. Due to its heterogeneous nature and requirement of a clean magnesium surface, this initiation may be a critical step.^[25]^ While benzyl chloride is highly reactive and readily forms the Grignard reagent even when cooled to 5 °C, bromobenzene takes some time and needs additional heating. Phenethyl chloride does not react without an additional activation reagent.
2. Further addition of organohalide in smaller amounts, after compensation of the initial reaction heat, until the formation rate of organomagnesium halide (e.g. ΔcPhEtMgCl) undercuts a predefined threshold. The piecemeal addition ensures an almost constant temperature within the reactor and the avoidance of significant over addition of the organohalide.
3. The addition of benzaldehyde, which was done in two consecutive sequences with decreasing amounts and is controlled by the ratio between Grignard and solvent avoiding addition of excess benzaldehyde.

The resulting peak areas per species of a synthesis run including control decision variables are shown in Figure 3 while the results of conducted experiments are shown in Table 1.

**Figure 3:**
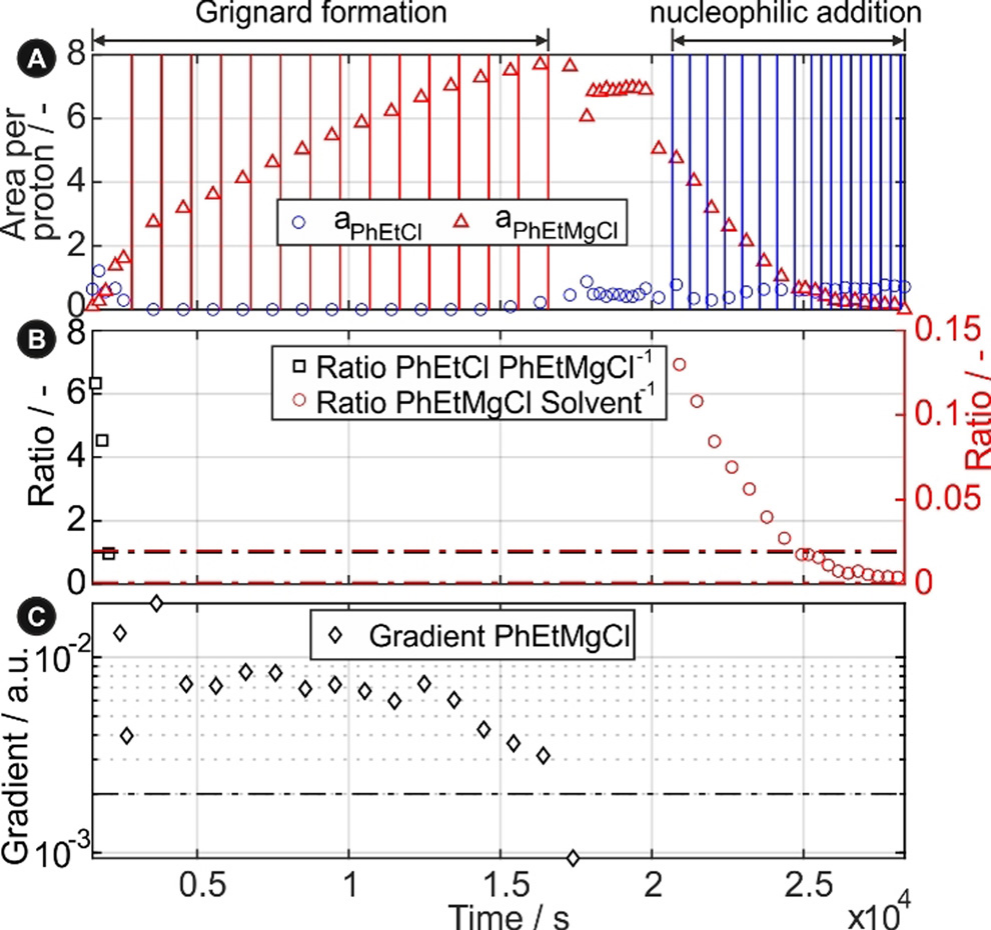
Experimental data of 1,3‐Diphenylpropan‐1‐ol synthesis run showing: A) proton‐normed species areas during whole experimentation including addition of PhEtCl (red, vertical) and Benzaldehyde (blue, vertical); B) area ratios which are used for control decision during the run with related thresholds (dotted, horizontal); C) gradient which is used for control decision during the run with related thresholds (dotted, horizontal).

**Table 1:** Results of the conducted experiments, including purity by qNMR, yield based on mass of Mg and initiation time.

Synthesis	Controlled	Purity^[b]^	Yield (calculated)	Initiation Time^[c]^	Benzaldehyde Consumption
Diphenylmethanol	No	61.2±2.2	9.61 g, 51 %	15 min	50 mL
Diphenylmethanol	Yes	83.0±1.7	15.74 g, 83 %	8.26 min	40 mL
Diphenylmethanol ^[a]^	Yes	80.0±2.9	7.07 g, 62 %	7.22 min	26 mL
Diphenylmethanol ^[a]^	Yes	78.9±1.9	8.09 g, 71 %	12.45 min	32 mL
1,2‐Diphenylethanol	No	84.8±0.0	15.14 g, 74 %	15 min	60 mL
1,2‐Diphenylethanol	Yes	89.3±0.1	13.19 g, 65 %	3.35 min	39 mL
1,2‐Diphenylethanol ^[a]^	Yes	68.2±0.0	6.67 g, 54 %	15 min	21 mL
1,2‐Diphenylethanol ^[a]^	Yes	72.3±0.1	5.71 g, 47 %	3.36 min	17 mL
1,3‐Diphenylpropan‐1‐ol	No	91.1±0.1	18.38 g, 84 %	15 min	50 mL
1,3‐Diphenylpropan‐1‐ol	Yes	83.2±0.0	19.23 g, 88 %	7.20 min	50 mL
1,3‐Diphenylpropan‐1‐ol	Yes	91.7±0.0	18.96 g, 87 %	26 min	48 mL

[a] Amount to Mg lowered to 60 %. [b] Specified by ^1^H‐NMR (see Supporting Information). [c] Initiation time fixed for uncontrolled experiments

In summary, an analytical module providing compatibility with any online PAT (compact NMR instrument in our case) was successfully developed as extension for an existing automated synthesis platform. The availability of qualitative and quantitative real‐time data on present species enabled the application of simple feedback control. Hence, proceeding of the synthesis based on current species ratios instead of hard‐coded waiting times was implemented enabling potentially higher productivity of the synthesis platform. As a proof of concept, the Grignard reaction was chosen due to its relevance in synthesis and challenging analytical conditions involving solids. The measurement and evaluation of NMR spectra was successfully performed during the whole synthesis. It proved capable of handling highly solids‐laden liquid resulting in reduced spectra quality. In contrast to NMR, optical spectroscopy often fails when solid particles are present and hence, the optical properties of the probe are widely varying compared to a pure liquid.

In conclusion, we show the replaceability of hard‐coded amounts by a self‐adjusting, feedback‐controlled approach. With little knowledge about the expected yields of the previous transformations in multi‐step synthesis, the maximum amounts to be added can be defined, improving performance in such hard to control biphasic systems. Optimized synthesis runs, resulting in a reduction of the amounts of starting material needed thus showed less reagent consumption. Therefore, the use of PAT leads to an economic benefit, increasing the flexibility of the synthesis platform, and such improvements are going to be vital for the automatic multi‐stage synthesis procedures. Adjusting the amounts based on intermediate yields reduces reagent consumption and improves efficiency, overall costs and purity. Further applications include the identification of unwanted by‐products as well as lowering the amount of by‐product produced. The modular approach also allows for an easy implementation of further PAT enabling supplementary methods or on‐the‐run calibrations. Future work will include optimization using the Chemputer with the XDL language so that XDLs can be versioned and hence used on systems without PAT sensors. Also, the self‐adjusting approach will be improved by machine learning algorithms.

Palladium‐catalyzed cross‐coupling reactions are among the most powerful and well‐established class of organic transformations^[26]^ and therefore attractive reaction candidates to prove a broad application of the presented method. In particular, the Suzuki–Miyaura cross‐coupling of aryl halides with organoboron compounds is widely applied.^[27]^ Merging the herein presented work with the automated iterative cross coupling of N‐methyliminodiacetic acid (MIDA) boronates developed by Burke and co‐workers^[7f]^ and already demonstrated on the Chemputer by Cronin and co‐workers^[16b]^ would vastly expand the applicability and prove the modular concept of both, the Chemputer and the attached analytical module. The progress of deprotection of the MIDA boronate could be assessed and the end point in the coupling step could be determined. More work will be necessary to guarantee an oxygen‐free and dry atmosphere during sampling and further improve reliable handling of reaction suspensions containing solid base and catalyst within the analytical module. In case that possible, analytical challenges like missing NMR visibility of certain species and severe signal overlapping are carefully considered, the presented development is applicable to a wide variety of chemical reactions, thus further paving the way towards a digitalized lab.

## Conflict of interest

The authors declare no conflict of interest.

## Supporting information

As a service to our authors and readers, this journal provides supporting information supplied by the authors. Such materials are peer reviewed and may be re‐organized for online delivery, but are not copy‐edited or typeset. Technical support issues arising from supporting information (other than missing files) should be addressed to the authors.

Supporting Information
