# Supplementary material for: Standardization and Control of Grignard Reactions in a Universal Chemical Synthesis Machine using online NMR
Source: Angew Chem Int Ed Engl. 2021 Aug 6;60(43):23202–6. doi: 10.1002/anie.202106323 (PMC8597166; doi:10.1002/anie.202106323)
Supplement: Supplementary file 1 — Supporting Information [file ANIE-60-23202-s001.pdf]

Angewandte Chemie
Eine Zeitschrift der Gesellschaft Deutscher Chemiker

Supporting Information

Standardization and Control of Grignard Reactions in a Universal Chemical Synthesis Machine using online NMR

Martin Bornemann-Pfeiffer[+], Jakob Wolf[+], Klas Meyer, Simon Kern, Davide Angelone, Artem Leonov, Leroy Cronin, and Franziska Emmerling**

anie_202106323_sm_miscellaneous_information.pdf

Supporting Information

Table of Contents

Table of Contents 1
Experimental Procedures 2
1. Materials and Methods 2
2. Automated Procedure 3
2.1 Development of Control 5
2.2 Activation of Magnesium Grit 5
2.3 Synthesis of Diphenylmethanol 6
2.4 Synthesis of Diphenylethanol 6
2.5 Synthesis of 1,3-Diphenylpropan-1-ol 6
3. Results and Discussion 7
3.1 Synthesis of Diphenylmethanol, non-controlled 7
3.2 Synthesis of Diphenylmethanol, controlled 9
3.3 Synthesis of 1,2-Diphenylethanol, non-controlled 12
3.4 Synthesis of 1,2-Diphenylethanol, controlled 14
3.5 Synthesis of 1,3-Diphenylpropan-1-ol, non-controlled 17
3.6 Synthesis of 1,3-Diphenylpropan-1-ol, controlled 19
3.7 Heat of reaction 21
References 21

Experimental Procedures

1. Materials and Methods

Automation platform

As automation platform, the "Chemputer" was used as built and published by the Cronin Group, including following software modules: Chempiler (version 2.0.9), ChemputerAPI (version 2.0), SerialLabware (version 1.1.1), AnalyticalLabware (version 0.1). Also, in line with [1,2], the Chemputer was equipped with an IKA RCT digital stirring plate (reflux module), a Heidolph HeiTorque 100 overhead stirrer (liquid-liquid-separation module), an IKA RV10 rotary evaporator plus heating bath HB10, a Julabo CF41 recirculation chiller with Snowstorm (Drysyn) adapter and an inert gas system[2]

Automation Software for feedback control (available from the authors upon request) was written in Python (3.7), with the modules mySQL-python (version 1.2.5) and python-opcua (version 0.98.12) downloaded from pypi.

Chemicals

All chemicals and solvents were used as acquired from commercial suppliers, without further purification: Benzyl chloride (Merck), Phenethyl chloride (Alfa Aesar, 100g), Benzaldehyde (Roth, >= 99.5%), Bromobenzene (Sigma Aldrich, 100g), Diethylether (ChemSolute, 2.5 L) and Magnesium grit (Sigma Aldrich, 250g).

Process NMR instrument

During chemical synthesis a compact NMR (Spinsolve Carbon Ultra, Magritek, Aachen, Germany) operating at 43.72 MHz capable of measuring ^{1}H, ^{19}F and ^{13}C nuclei was employed as analytical instrument acquiring 4 proton scans with 90° pulse angle and 52.8 s overall acquisition time. The acquired spectra were automatically saved.

Spectra Processing

New spectra were identified via folder monitoring and evaluated by PEAXACT ProcessLink (S-Pact Gmbh, Aachen, Germany). The specific mixture models consisting of pure component hard models were created and parametrized prior to experimentation based on pure component spectra.

Product Characterization

High-resolution NMR spectra of the crude reaction products after aqueous workup were acquired using a 500 MHz NMR spectrometer (VNMRS 500 equipped with a OneNMR 5 mm probe, Varian Associates, Palo Alto, CA, USA). For quantitative NMR experiments in-house internal standard material Trimethyl-1,3,5-benzoltricarboxylate (purity by qNMR 99.9 %) was used. Gravimetric operations were performed on an ultra-microbalance (XP2 U/M, Mettler-Toledo GmbH, Gießen, Germany). Relaxation delay was set to $>7*T_1$ to ensure quantitative measurement conditions.

Temperature Sensor

For measuring internal reactor temperature, a PT100 temperature probe connected to an IKA RCT digital stirring plate (IKA, Staufen, Germany) was used, readings taken every 15 seconds and stored in the SQL, together with a timestamp. The probe was immersed in a thin NMR glass tube filled with paraffin oil which again was fitted into the reaction flask with a custom-made PTFE stopper with suitable bore.

Syringe Inlet

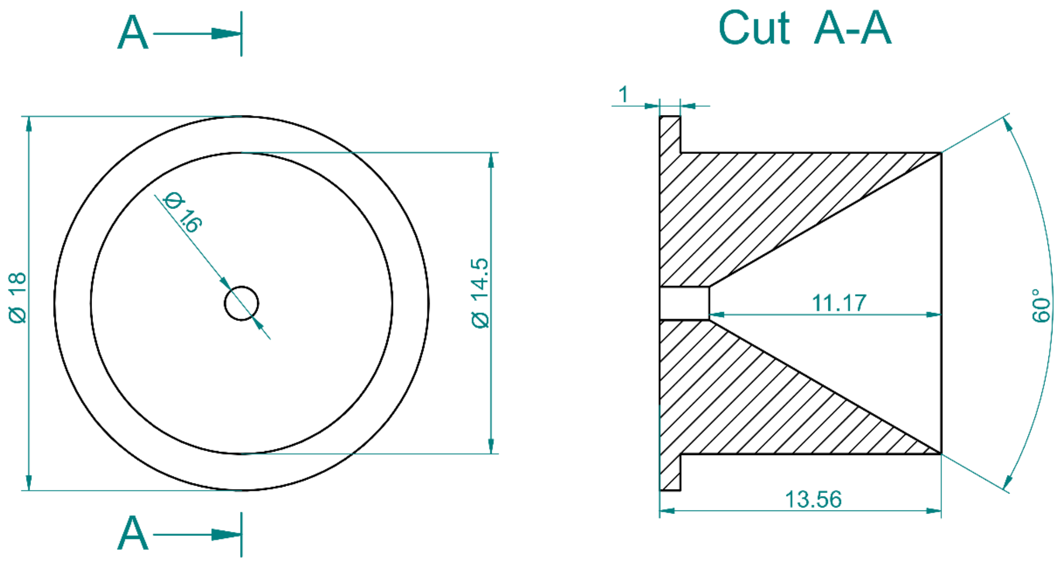

Figure 1: Construction Drawing of the custom-made inlet for syringes made from PTFE, reducing blockages when pumping suspensions

2. Automated Procedure

The automated, experimental procedure for the Grignard reaction was adopted from Steiner et al. [1], with small variations and the addition of feedback control and analytical measurements. A 4-necked round-bottom flask was equipped with a stirring bar and a water-cooled reflux condenser connected to a stream of dry argon. Two ground-glass joints with a GL14 thread were connected to PTFE tubing (1.6 mm ID) with a PTFE inlet and cap. One piece of tubing was connected to the Chemputer backbone, while the other was routed through the NMR probehead ending in a glass syringe (ILS, Germany) with custom-made PTFE bottom part (see **Figure 1**), actuated by a syringe pump (Gemini88, KDScientific, USA). Care was taken to not overly constrict the tubing by avoiding tight bends to prevent blockage and to guide the tubing through the NMR magnet as straight as possible. For modification of the glass syringe with the custom outlet, the syringe was refluxed in toluene, which dissolved the glue and then reglued with the custom part in place using epoxy glue (UHU Plus Endfest 2K, Uhu, Germany). The third glass joint was used to insert a Pt100 thermocouple into the reactor. Cooling and heating of the reactor was achieved by a recirculation chiller (Julabo, Germany) fitted to a cooling adapter (SnowStorm, Drysyn, UK).

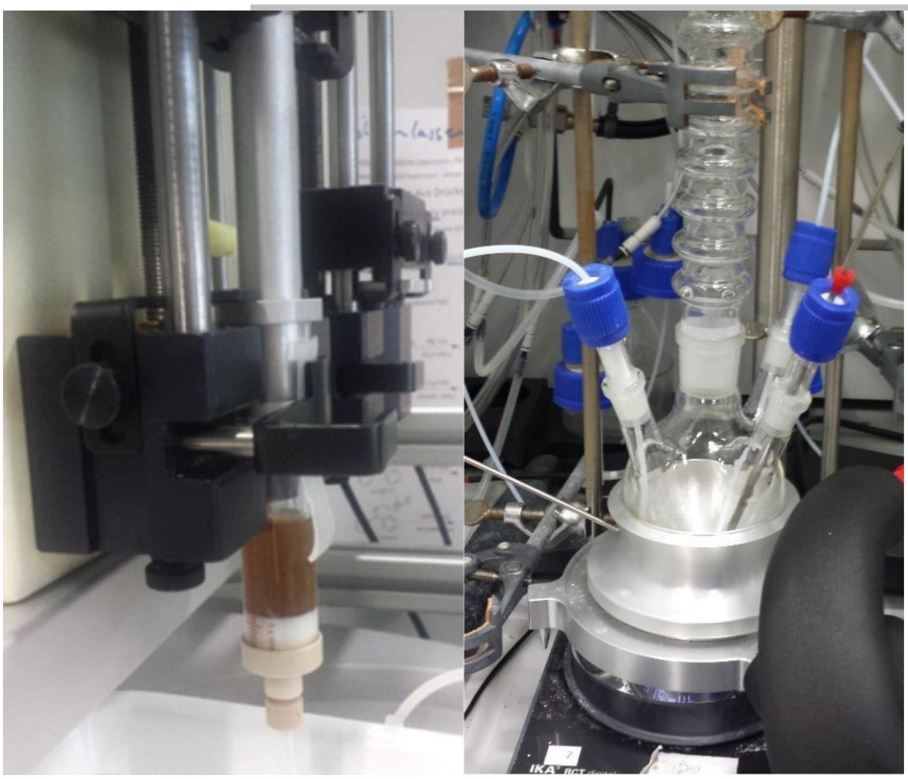

Figure 2: Photographs of the experimental setup. Left: Syringe for taking reaction solution samples with custom-manufactured, funnel-shaped PTFE outlet (see **Figure 1**); Right: 4-necked flask with PT100 Thermocouple in glass tube and sampling tubing.

The peak areas (A_a), calculated for every species through Indirect Hard Modeling (IHM), are normalized through the number of protons per species ($n_{proton,a}$) resulting in relative concentrations ($c_{a,rel}$)(Eq. 1).

$$c_{a,rel} = \frac{A_a}{n_{proton,a}} \quad (1)$$

Based on these, two parameters can be calculated: First, the concentration gradient of one species ($\Delta c_{a,rel}$) in relation to the added voluminal (V_{add})(Eq. 2). Second, the ratio of selected species concentrations (x_a)(Eq. 3).

$$\Delta c_{a,rel} = \left| \frac{c_{a,rel}(t_n) - c_{a,rel}(t_{n-1})}{(t_n - t_{n-1}) * V_{add}} \right| \quad (2)$$

$$x_a = \frac{c_{a,rel}}{c_{b,rel}} \quad (3)$$

The applied synthesis procedure includes three feedback-controlled steps. First, the initiation of the organomagnesium halide formation, which underlies varying time spans due to its heterogeneous nature and different reactivity of the starting materials, is detected by the ratio of organohalide to Grignard reagent. This might be a critical step during Grignard synthesis as mentioned by Tilstam et al. [3]. After compensation of the initial reaction heat, further organohalide is added in smaller amounts until the formation rate of organomagnesium halide (e.g. $\Delta c_{PhEtMgCl}$) undercuts a predefined threshold. The piecemeal addition ensures an almost constant temperature within the reactor as well as the avoidance of significant overaddition of the organohalide. The addition of benzaldehyde was done in two consecutive sequences, the added amount was decreased in the second sequence to avoid excess addition. The completion of the reaction is indicated by the ratio of Grignard to solvent. After the reaction and upon acidic workup, excess magnesium is simply dissolved and excess Grignard reagent will react to the corresponding hydrocarbon, which is relatively volatile (benzene, toluene and ethylbenzene) and will be removed upon solvent removal. The main expected nonvolatile byproducts are biphenyl, bibenzyl and 1,4-Diphenylbutane by Wurtz coupling. In case of Phenethylmagnesiumbromide, Styrene is a possible byproduct by abstraction of a proton from Phenethylchlorid and elimination of Cl- or by hydride transfer to benzaldehyde. Except for the condensation products, and the starting materials, everything is expected to be removed with the solvent.
To compare uncontrolled (hardcode volume and times) and controlled reaction sequences, the yield was calculated based on the limiting reagent, magnesium. Also, the purity of the crude product was determined by qNMR. High purity mainly translates to low side-reaction and quantitative or sub-quantitative reagent additions, the latter would translate to low yield. This means that high yield and high purity indicate good performance of feedback control.

2.1 Development of Control

To investigate the formation of diphenylmethanol, automated reactions with online analytics, but without feedback control were performed. Reagents were added quantitatively, split in small portions and spectra measured after a defined delay with the NMR device. Based on the relative concentration of each species obtained by subsequent spectra evaluation via IHM, different approaches for decision making (**Figure 3**) were investigated. Since no calibration was involved, only relative concentrations were accessible which was sufficient in presented reaction surveillance. Absolute, quantitative results are easily accessible by performing a single-point calibration on one of the species. The two-phase reaction between Mg and organic halide and the absence of any information on the amount of solid Magnesium poses a challenge. The only way to determine the complete consumption of Mg is through overaddition of organic halide, thereby increasing impurities. A reduction of this excess can be achieved by reducing the organic halide volumes of the individual additions on cost of reaction time (sampling and measuring one proton NMR spectrum takes approx. 3 min). The reaction of available, solid Mg was tracked through the gradient of the resulting Grignard species. Further experiments showed that the ratio between the relative concentrations of Grignard and organohalide could be employed with comparable performance.

The analytical situation is different in the consecutive reaction between Grignard and benzaldehyde. Despite the evolution of solid in case of Diphenylmethanol synthesis the benzaldehyde addition can be tracked well, since the still available Grignard reagent can be evaluated. The comparison with a threshold value allowed for the decision whether sufficient benzaldehyde was added to consume all measurable Grignard in order to reduce traces of benzaldehyde in the final product. The added volume of benzaldehyde corresponds well with the initial amount of magnesium (2.5 g respective 1.5 g, see experimental procedures) in the reaction.

To broaden the scope of achievable Grignard reactions, two other organic halides in addition to bromobenzene were chosen, namely benzyl chloride and phenethyl chloride, the former highly reactive, the latter of low reactivity and requiring an additional activation reagent. Apart from cooling the Grignard formation in case of phenethylchloride and benzyl chloride, the underlying control concept (including applied threshold values) remained the same for all conducted reactions.

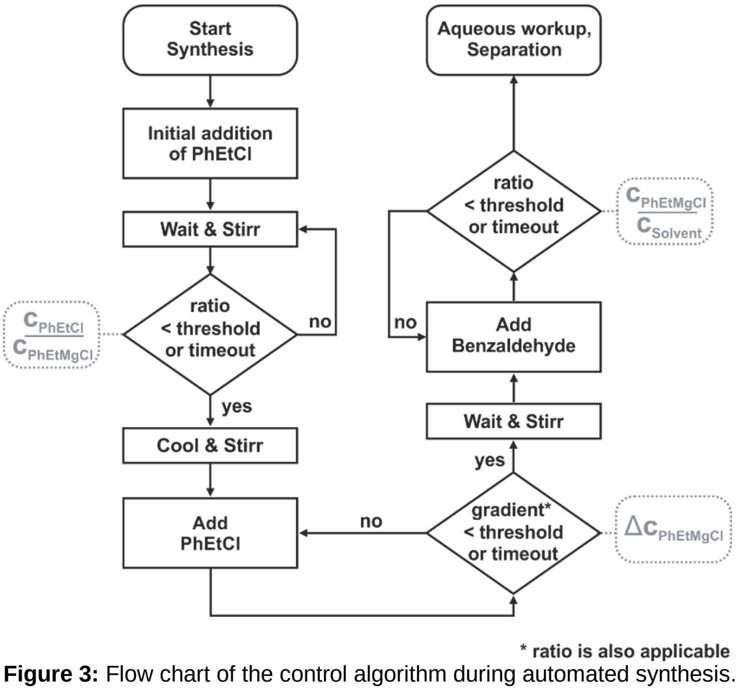

Figure 3: Flow chart of the control algorithm during automated synthesis.

2.2 Activation of Magnesium Grit

2.5g of magnesium grit (or 1.5g to test how the feedback algorithm copes with lower available amounts) (Sigma Aldrich), were weighed manually into the reactor. Under stirring (150 rpm) the reactor was heated to 150°C, while streaming dry argon over the magnesium. After 15 min, the reactor was tightly closed and passively cooled to room temperature. From here on, all actions were performed by the Chemputer.

2.3 Synthesis of Diphenylmethanol

30 mL of diethyl ether were added to the magnesium, followed by 2 mL of the bromobenzene and 10 mL of diethyl ether. The mixture was heated to reflux. Every three minutes, the reaction was sampled by low field NMR and actively cooled back to room temperature upon the presence of equal amounts of halide and Grignard species. 1 mL of bromobenzene was added. From here on, bromobenzene was added in portions of 1 mL and a sample taken one minute after addition. This was repeated until the gradient of phenylmagnesiumbromide fell below a certain threshold. After 15 min the mixture was heated to reflux. After further 15 min, a solution of benzaldehyde in diethyl ether (2 M, ~50 mL in total, depending on Grignard amount) was added slowly (1mL/min) and in portions of 5 mL, measuring an NMR spectrum after every addition. When the ratio between Grignard and solvent fell below a certain value, smaller portions of the aldehyde solution (1 mL) were added, until the ratio of Grignard to solvent went below a certain threshold. After further 3 hours of refluxing, the reaction was cooled down and quenched with 25 mL of water. 100 mL of aqueous hydrochloric acid (2 M) were added and the suspension stirred for 30 min. The now clear phases were separated, and the solvent removed from the organic phase under reduced pressure, which yielded the crude title compound. ^{1}H NMR:($CDCl_3$, 400 MHz) δ 2.16 (brs, 1H), 5.79 (s, 1H), 7.21-7.35 (m, 10H). Known substance, [4]

2.4 Synthesis of Diphenylethanol

30 mL of diethyl ether were added to the magnesium, the suspension cooled to 5 °C and 2 mL of benzyl chloride and further 10 mL of diethyl ether added. Every three minutes, the reaction was sampled by low field NMR. Upon the presence of equal amounts of halide and Grignard species, 1 mL of benzylchloride was added. From here on, benzylchloride was added in portions of 1 mL and a sample measured by NMR after 1 min. This was repeated until the gradient of benzyl magnesiumchloride fell below a certain threshold. After 15 min the mixture was heated to reflux. After further 15 min, a solution of benzaldehyde in diethyl ether (2 M, ~50 mL in total, depending on Grignard amount)) was added in portions and slowly, measuring an NMR spectrum after every addition. When the ratio between Grignard and solvent fell below a certain value, smaller portions of the aldehyde solution (1 mL) were added, until the ratio of Grignard to solvent went below a certain threshold. After further 3 hours of refluxing, the reaction was cooled down and quenched with 25 mL of water. 100 mL of aqueous hydrochloric acid (2 M) were added and the suspension stirred for 30 min. The now clear phases were separated, and the solvent removed from the organic phase under reduced pressure, which yielded the crude title compound. A sample was prepared and analyzed with ^{1}H NMR:($CDCl_3$, 400 MHz) δ 2.16 (brs, 1H), 2.95-3,05 (m, 2H), 4.85 (q, 1H), 7.16-7.34 (m, 10H). Known substance, [5]

2.5 Synthesis of 1,3-Diphenylpropan-1-ol

30 mL of diethyl ether were added to the magnesium, the suspension cooled to 5 °C and 2 mL of the phenethylchloride were added with further 10 mL of diethyl ether. 0.5 ml of phenethylbromide were added manually to start the reaction. Every three minutes, the reaction was sampled by low field NMR and actively heated to room temperature upon the presence of equal amounts of halide and Grignard species. 1 mL of phenethylchloride was added. From here on, phenethylchloride was added in portions of 1 mL and a sample taken after one min. This was repeated until the gradient of phenylethyl magnesiumchloride fell below a certain threshold. After 15 min the mixture was heated to reflux. After further 15 min, a solution of benzaldehyde in diethyl ether (2 M) was added slowly in portions of 5 mL, measuring an NMR spectrum after every addition. When the ratio between Grignard and solvent fell below a certain value, smaller portions of the aldehyde solution (1 mL) were added, until the ratio of Grignard to solvent went below a certain threshold. After further 3 hours of refluxing, the reaction was cooled down and quenched with 25 mL of water. 100 mL of aqueous hydrochloric acid (2 M) were added and the suspension stirred for 30 min. The now clear phases were separated, and the solvent removed from the organic phase under reduced pressure, which yielded the crude title compound. ^{1}H NMR: ($CDCl_3$, 400 MHz) δ 1.93 (brs, 1H), 1.98-2.16 (m, 2H), 2.63-2.77 (m, 2H), 4.66-4.69 (m, 1H), 7.15-7.36 (m, 10H). Known substance, [6]

3. Results and Discussion

3.1 Synthesis of Diphenylmethanol, non-controlled

The acquired spectra during the synthesis of Diphenylmethanol without feedback control are subsequently shown. In addition, high-resolution NMR spectra, acquired after the aqueous workup, are shown as well.

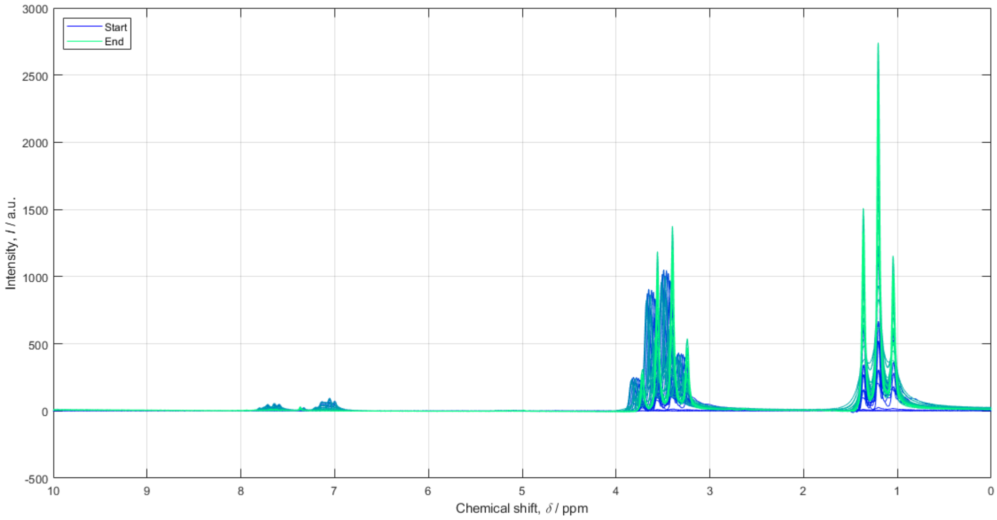

Figure 4: Superimposed ^{1}H-NMR spectra acquired with 43 MHz compact NMR instrument during uncontrolled chemical synthesis of Diphenylmethanol

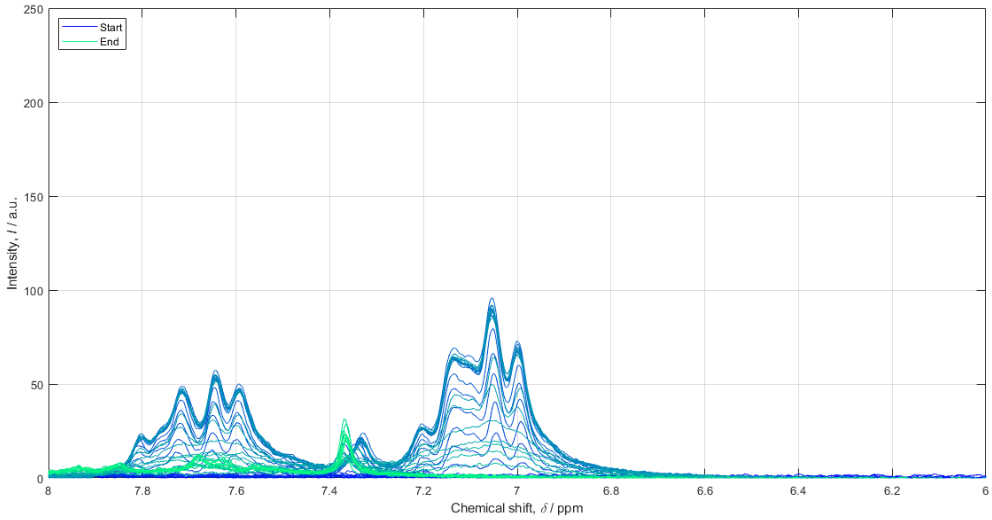

Figure 5: Superimposed ^{1}H-NMR spectra acquired with 43 MHz compact NMR instrument during chemical synthesis; close-up of aromatic region

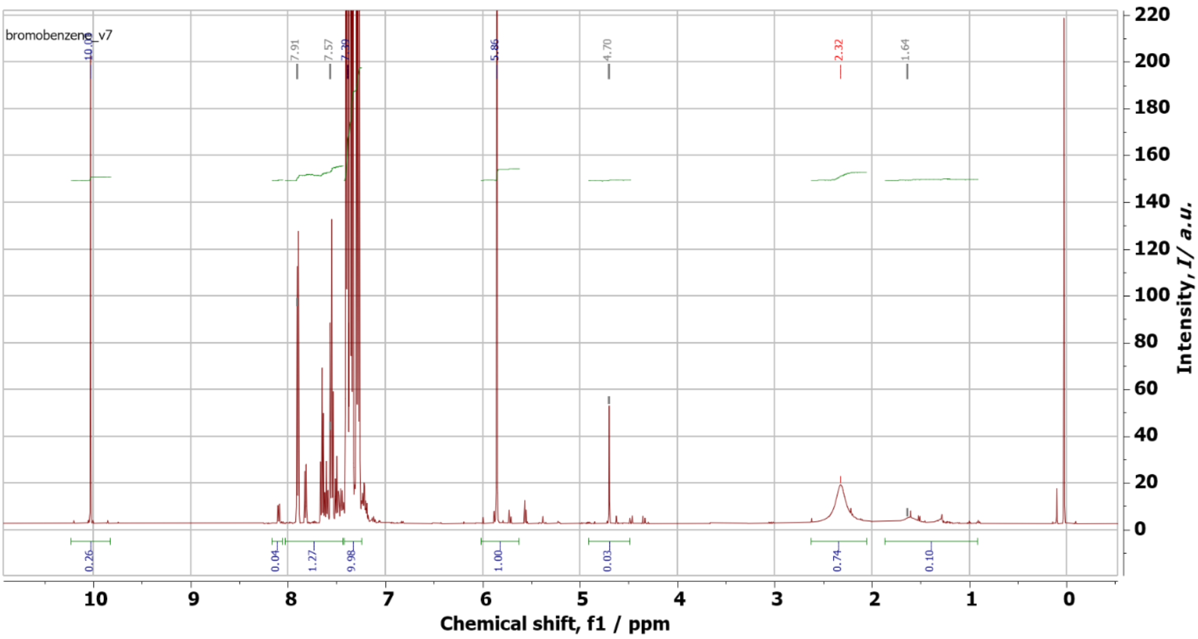

Figure 6: ^{1}H-NMR spectrum of crude reaction product of Diphenylmethanol synthesis acquired with 500 MHz NMR instrument.

3.2 Synthesis of Diphenylmethanol, controlled

The acquired spectra during the feedback-controlled synthesis of Diphenylmethanol and the applied hard models are subsequently shown. In addition, high-resolution NMR spectra, acquired after the aqueous workup, are shown as well.

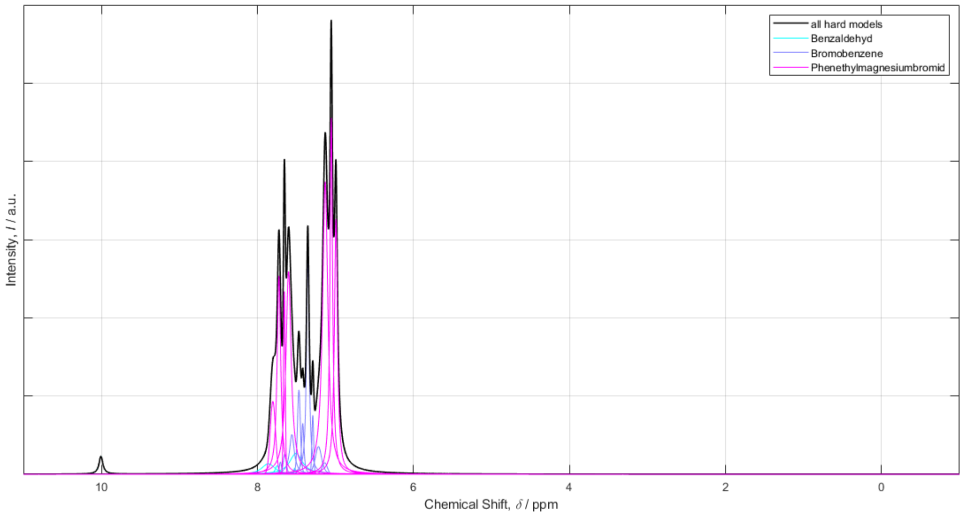

Figure 7: Underlying hard models for IHM-based mixture deconvolution during feedback controlled synthesis of Diphenylmethanol based on ^{1}H-NMR spectra, acquired with 43 MHz compact NMR instrument.

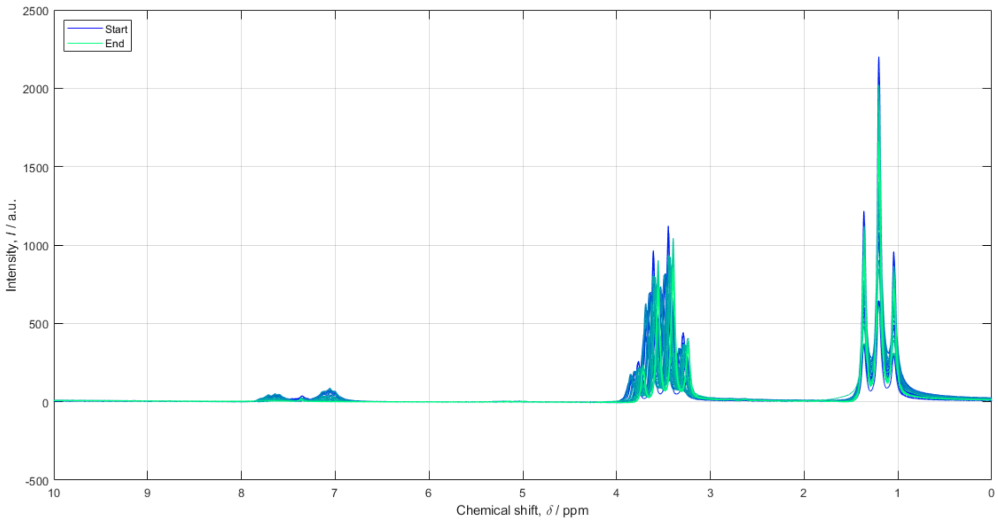

Figure 8 Superimposed ^{1}H-NMR spectra acquired with 43 MHz compact NMR instrument during feedback controlled chemical synthesis of Diphenylmethanol.

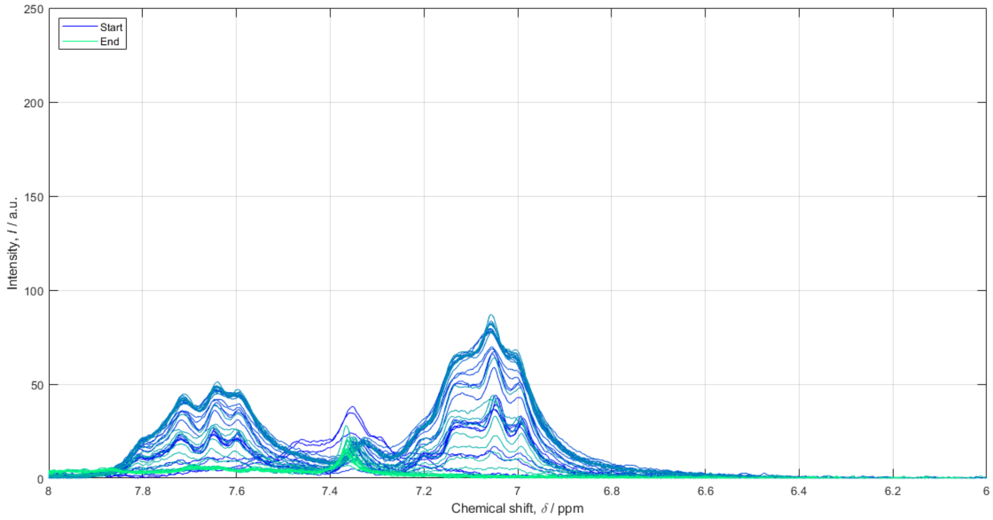

Figure 9 Superimposed ^{1}H-NMR spectra acquired with 43 MHz compact NMR instrument during feedback controlled chemical synthesis of Diphenylmethanol, close-up of aromatic region.

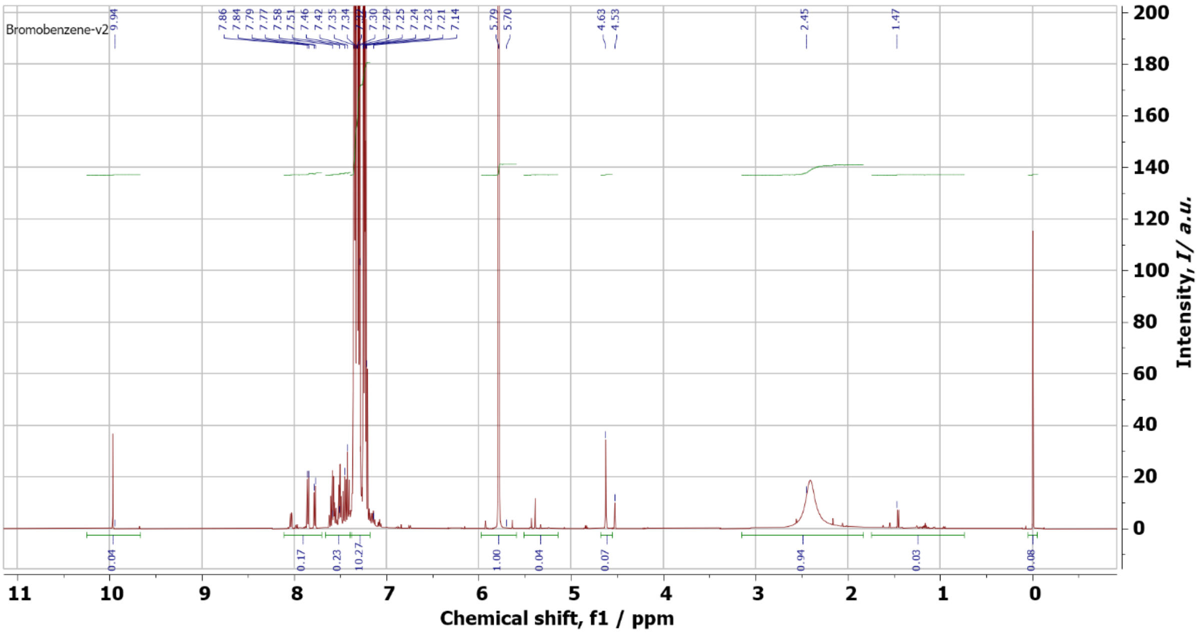

Figure 10: ^{1}H-NMR spectrum of crude reaction product of Diphenylmethanol synthesis with feedback control acquired with 500 MHz NMR instrument.

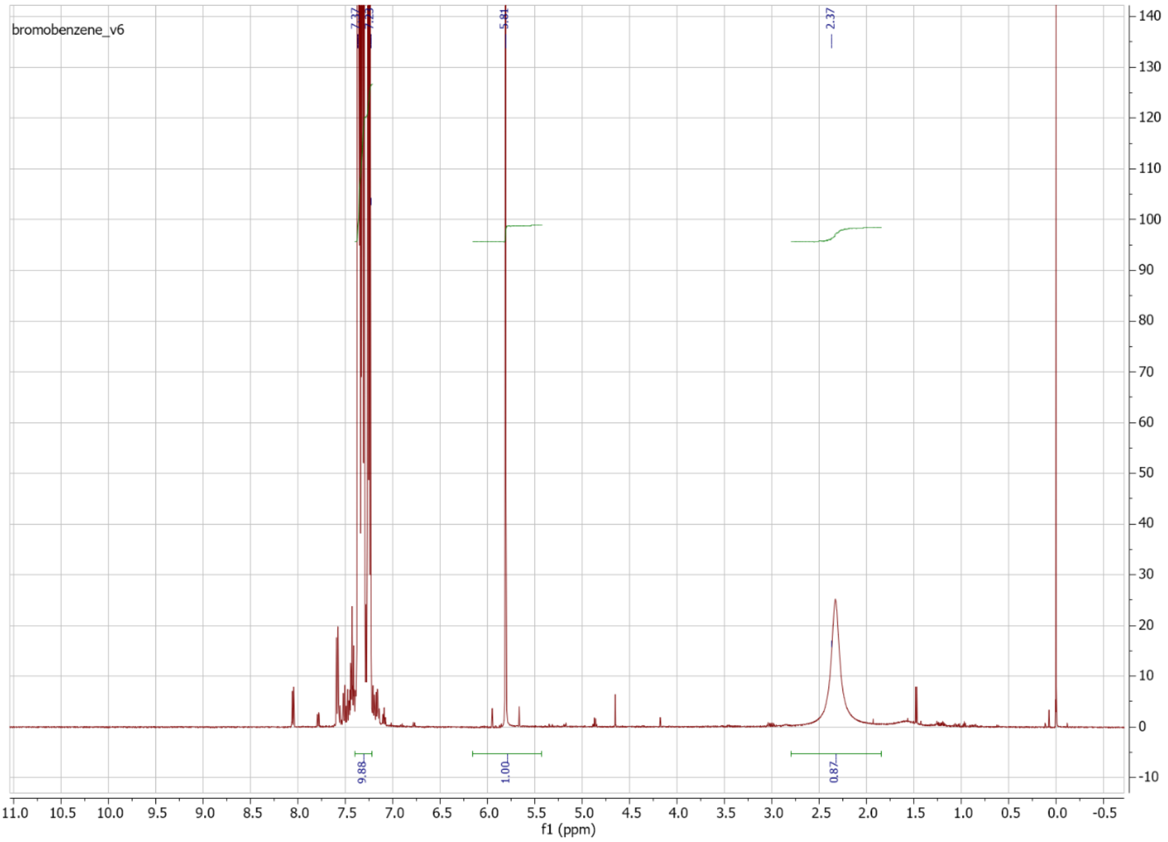

Figure 11: ^{1}H-NMR spectrum of crude reaction product of Diphenylmethanol synthesis with feedback control and reduced Mg amount acquired with 500 MHz NMR instrument.

3.3 Synthesis of 1,2-Diphenylethanol, non-controlled

The acquired spectra during the synthesis of 1,2-Diphenylethanol without feedback control are subsequently shown. In addition, high-resolution NMR spectra, acquired after the aqueous workup, are shown as well.

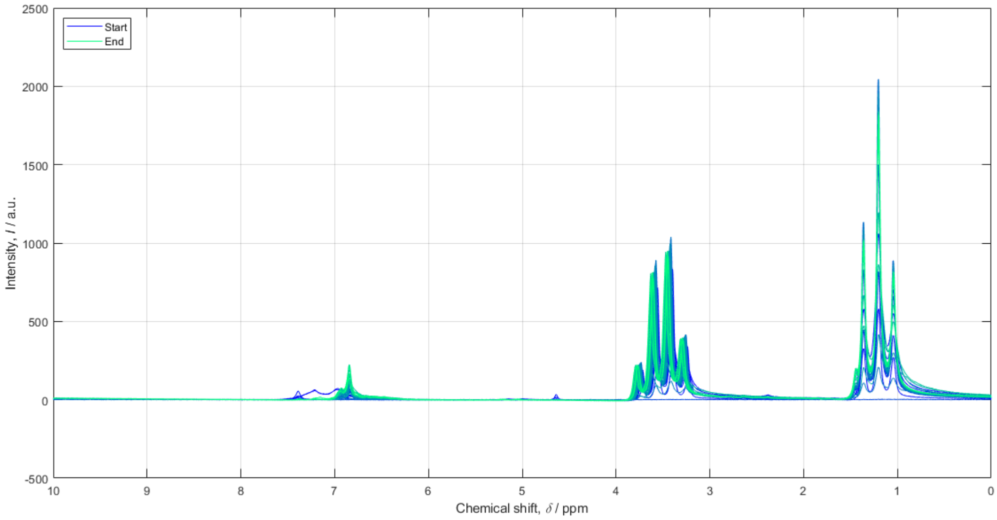

Figure 12: Superimposed ^{1}H-NMR spectra acquired with 43 MHz compact NMR instrument during uncontrolled chemical synthesis of 1,2-Diphenylethanol.

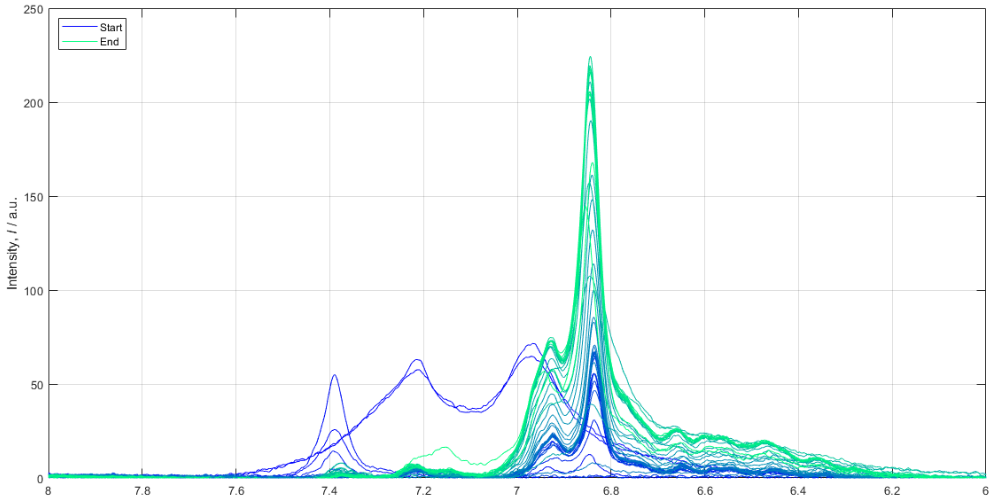

Figure 13: Superimposed ^{1}H-NMR spectra acquired with 43 MHz compact NMR instrument during uncontrolled chemical synthesis of 1,2-Diphenylethanol; close-up of aromatic region.

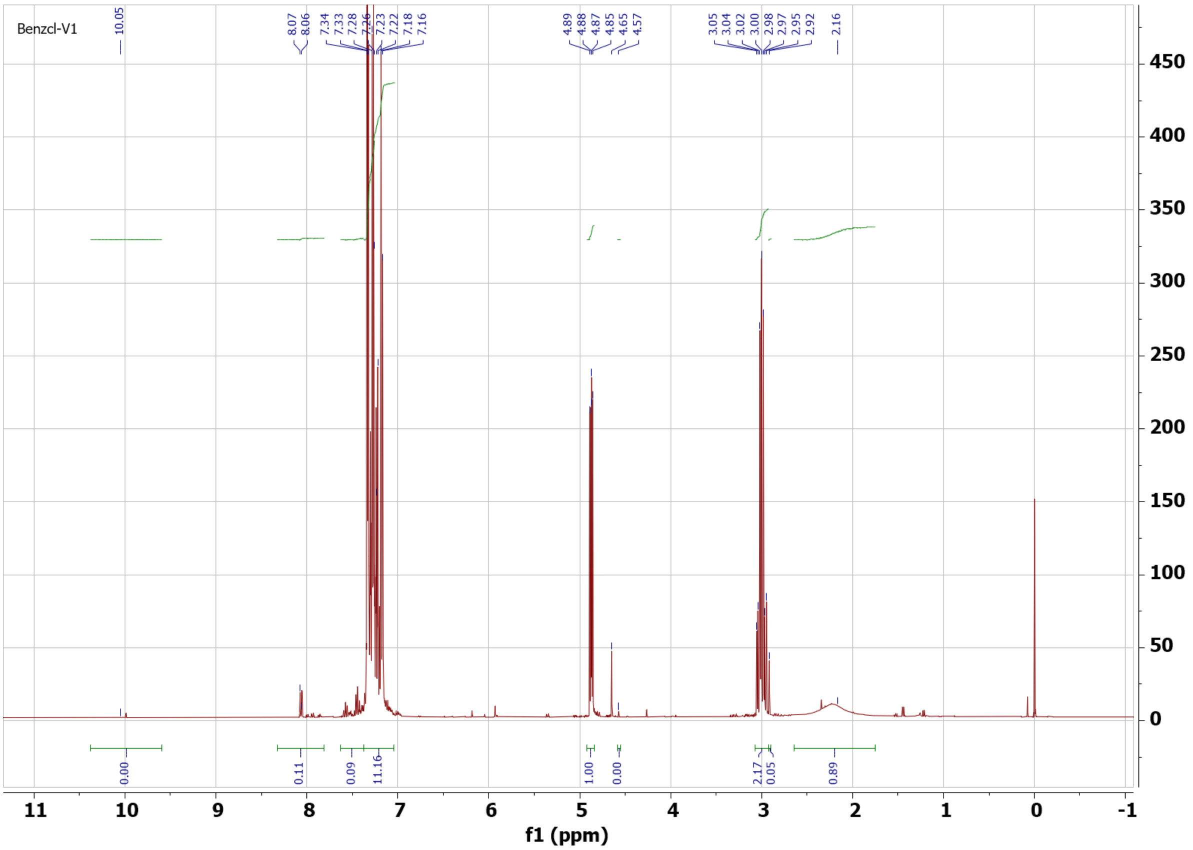

Figure 14 ^{1}H-NMR spectrum of crude reaction product of uncontrolled 1,2-Diphenylethanol synthesis acquired with 500 MHz NMR instrument.

3.4 Synthesis of 1,2-Diphenylethanol, controlled

The acquired spectra during the feedback-controlled synthesis of 1,2-Diphenylethanol and the applied hard models are subsequently shown. In addition, high-resolution NMR spectra, acquired after the aqueous workup, are shown as well.

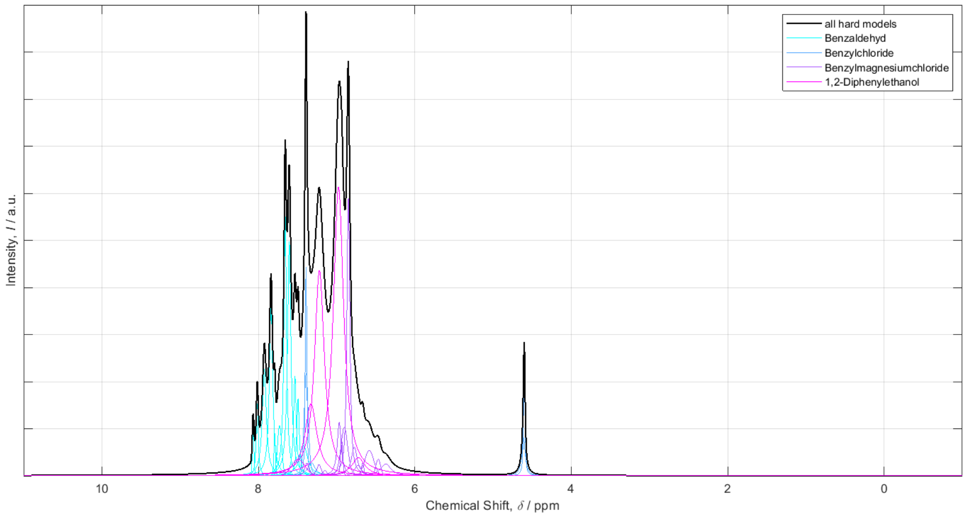

Figure 15: Underlying hard models for IHM-based mixture deconvolution during feedback controlled synthesis of 1,2-Diphenylethanol based on ^{1}H-NMR spectra, acquired with 43 MHz compact NMR instrument.

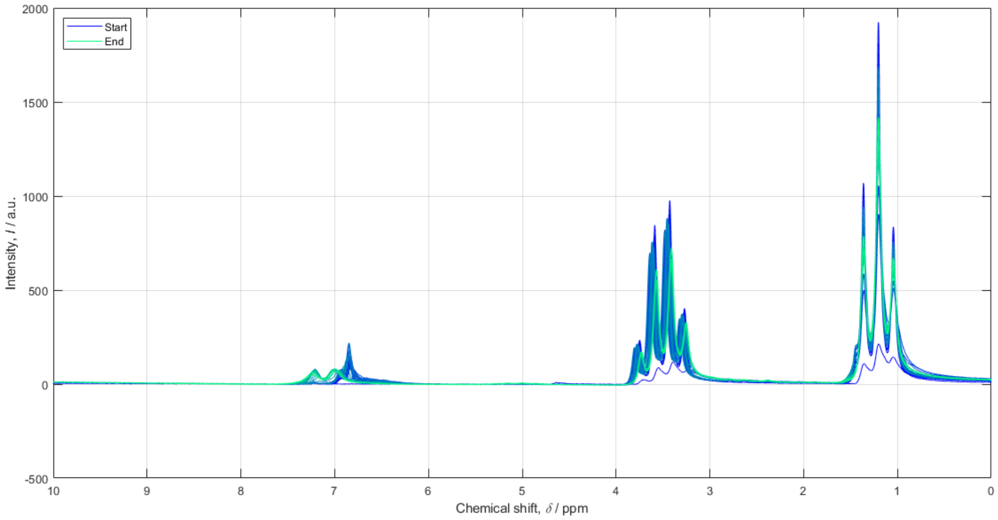

Figure 16: Superimposed ^{1}H-NMR spectra acquired with 43 MHz compact NMR instrument during feedback controlled chemical synthesis of 1,2-Diphenylethanol.

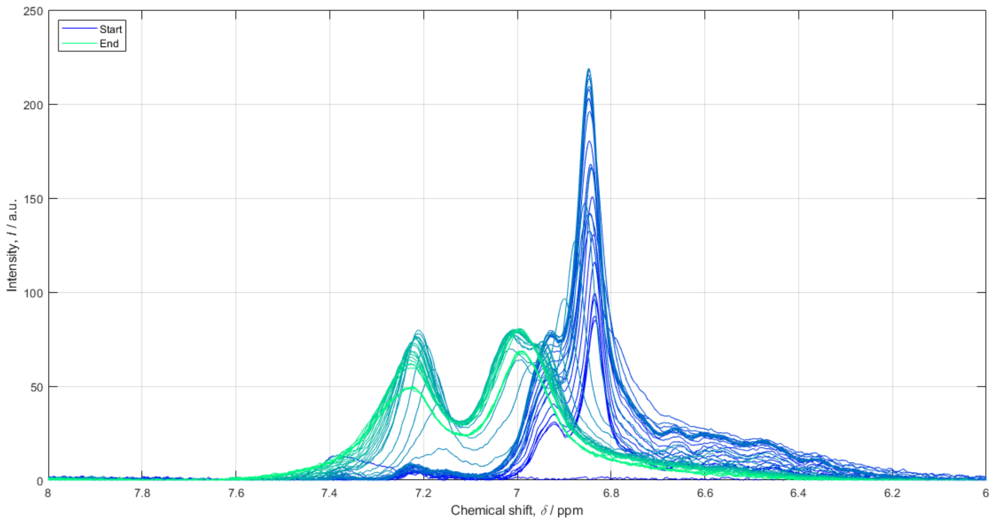

Figure 17: Superimposed ^{1}H-NMR spectra acquired with 43 MHz compact NMR instrument during feedback controlled chemical synthesis of 1,2-Diphenylethanol. Closeup of the aromatic region.

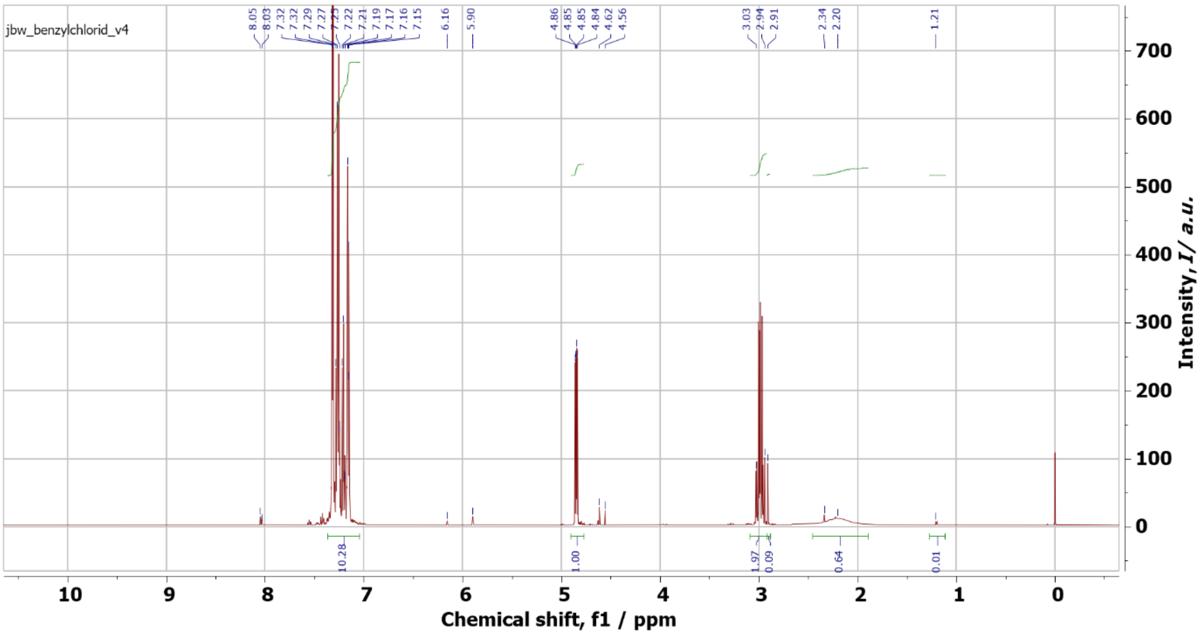

Figure 18: ^{1}H-NMR spectrum of crude reaction product of 1,2-Diphenylethanol synthesis with feedback control, acquired with 500 MHz NMR instrument.

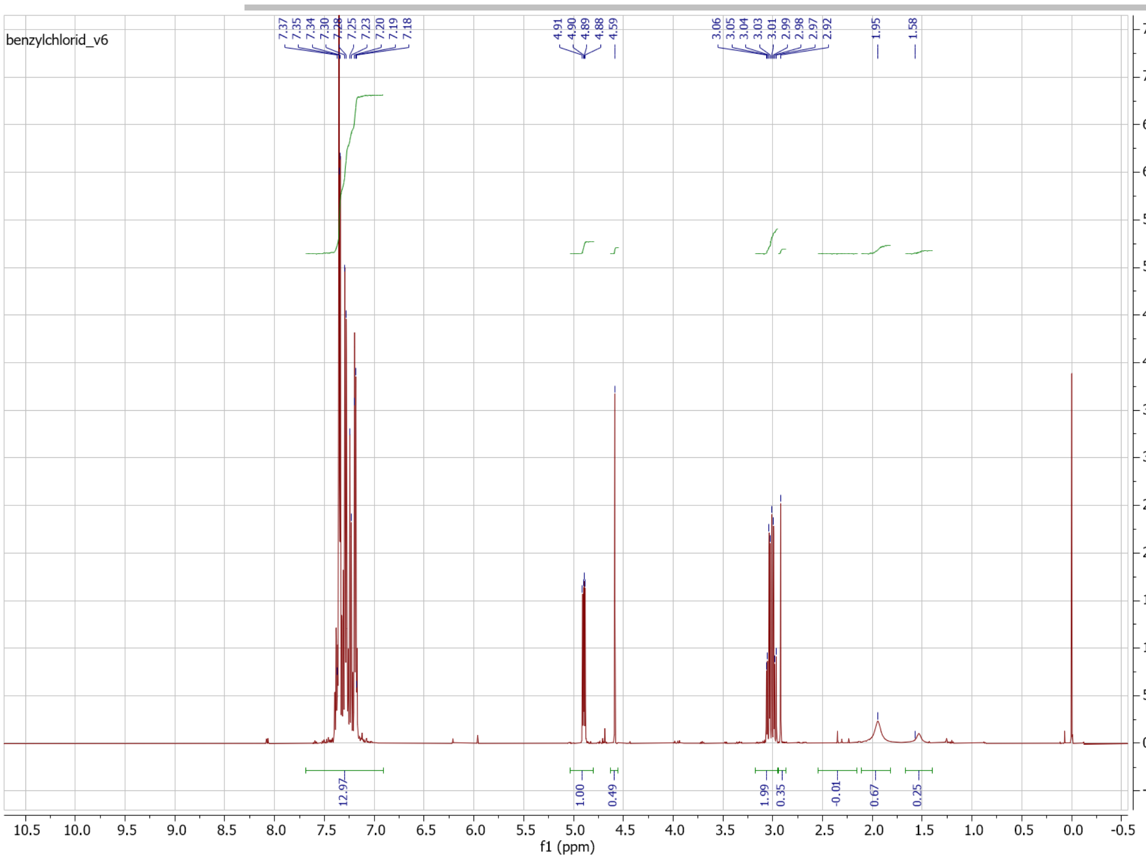

Figure 19: ^{1}H-NMR spectrum of crude reaction product of 1,2-Diphenylethanol synthesis with feedback control and reduced Mg amount acquired with 500 MHz NMR instrument.

3.5 Synthesis of 1,3-Diphenylpropan-1-ol, non-controlled

The acquired spectra during the synthesis of 1,3-Diphenylpropan-1-ol without feedback control are subsequently shown. In addition, high-resolution NMR spectra, acquired after the aqueous workup, are shown as well.

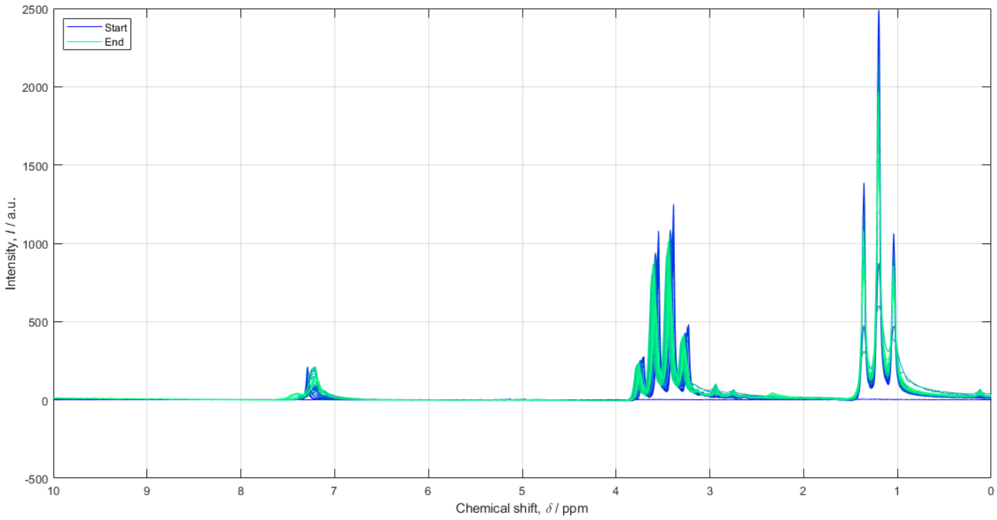

Figure 20: Superimposed ^{1}H-NMR spectra acquired with 43 MHz compact NMR instrument during uncontrolled chemical synthesis of 1,3-Diphenylpropan.1-ol.

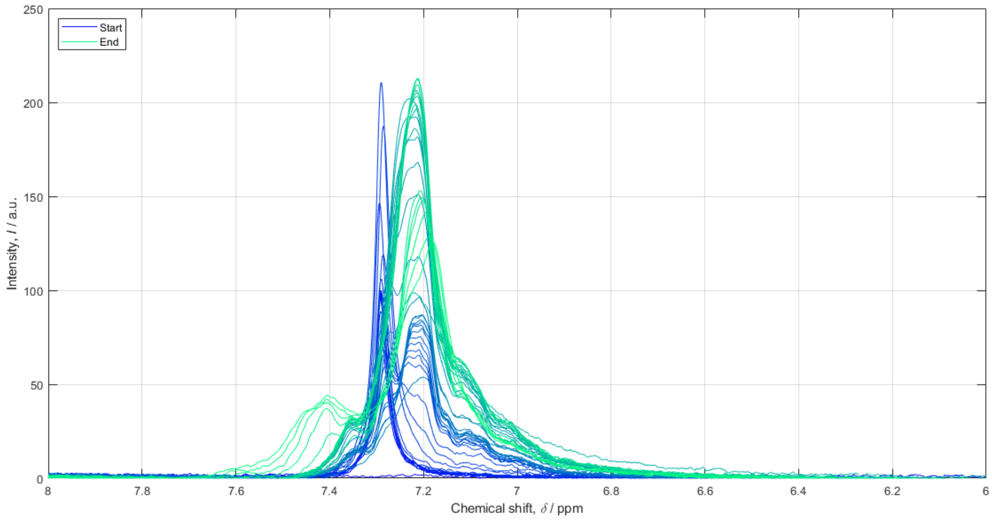

Figure 21: Superimposed ^{1}H-NMR spectra acquired with 43 MHz compact NMR instrument during uncontrolled chemical synthesis of 1,3-Diphenylpropan-1-ol; close-up of aromatic region.

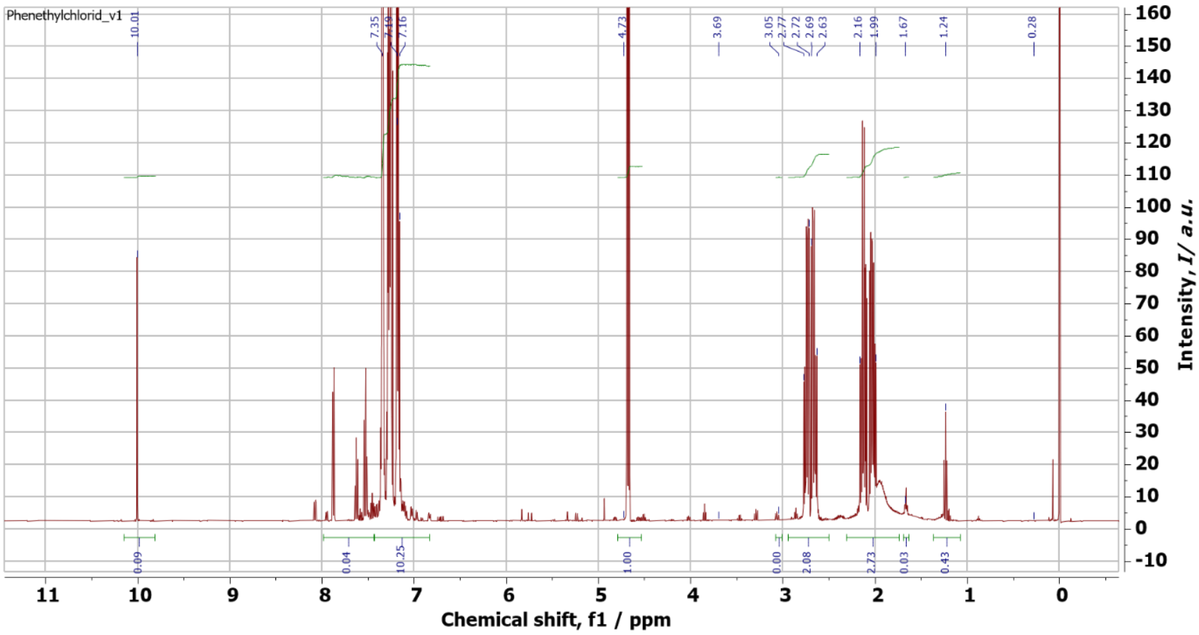

Figure 22: ^{1}H-NMR spectrum of crude reaction product of 1,3-Diphenyl-propan-1-ol synthesis acquired with 500 MHz NMR instrument. Excess benzaldehyde was manually added to verify quantitative reaction.

3.6 Synthesis of 1,3-Diphenylpropan-1-ol, controlled

The acquired Spectra during the feedback-controlled synthesis of 1,3-Diphenylpropan-1-ol and the applied hard models are subsequently shown. In addition, high-resolution NMR spectra, acquired after the aqueous workup, are shown as well.

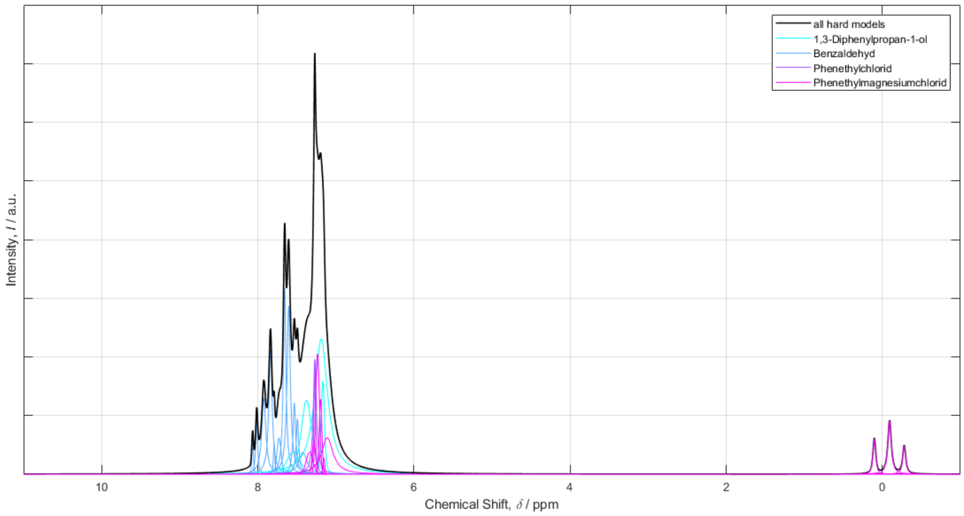

Figure 23: Underlying hard models for IHM-based mixture deconvolution during feedback controlled synthesis of 1,3-Diphenylpropan-1-ol based on ^{1}H-NMR spectra, acquired with 43 MHz compact NMR instrument.

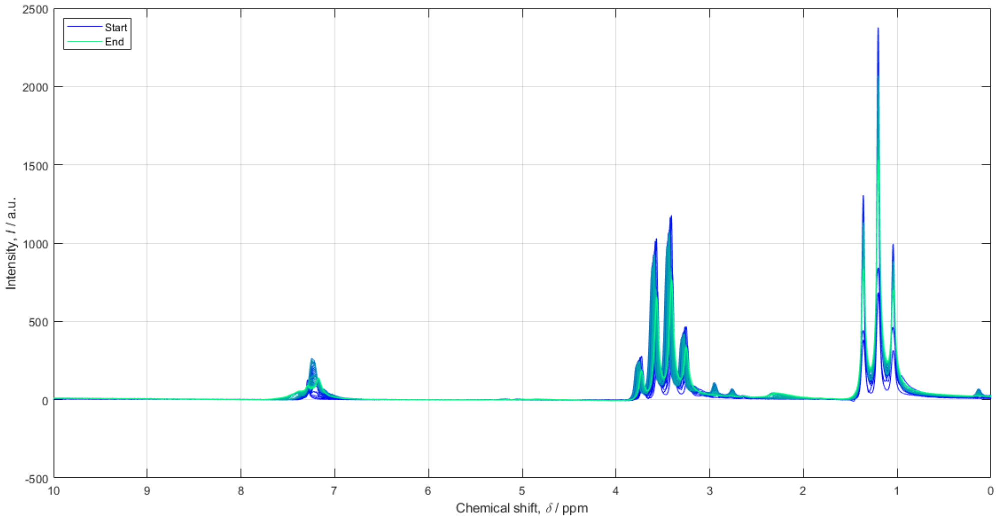

Figure 24: Superimposed ^{1}H-NMR spectra acquired with 43 MHz compact NMR instrument during feedback controlled chemical synthesis of 1,3-Diphenylpropan-1-ol.

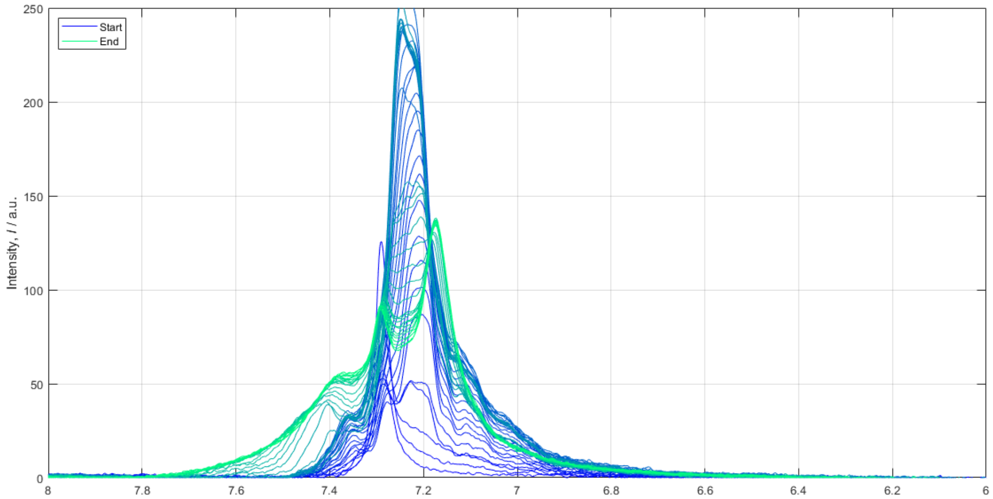

Figure 25: Superimposed ^{1}H-NMR spectra acquired with 43 MHz compact NMR instrument during feedback controlled chemical synthesis of 1,3-Diphenylpropan-1-ol; close-up of aromatic region.

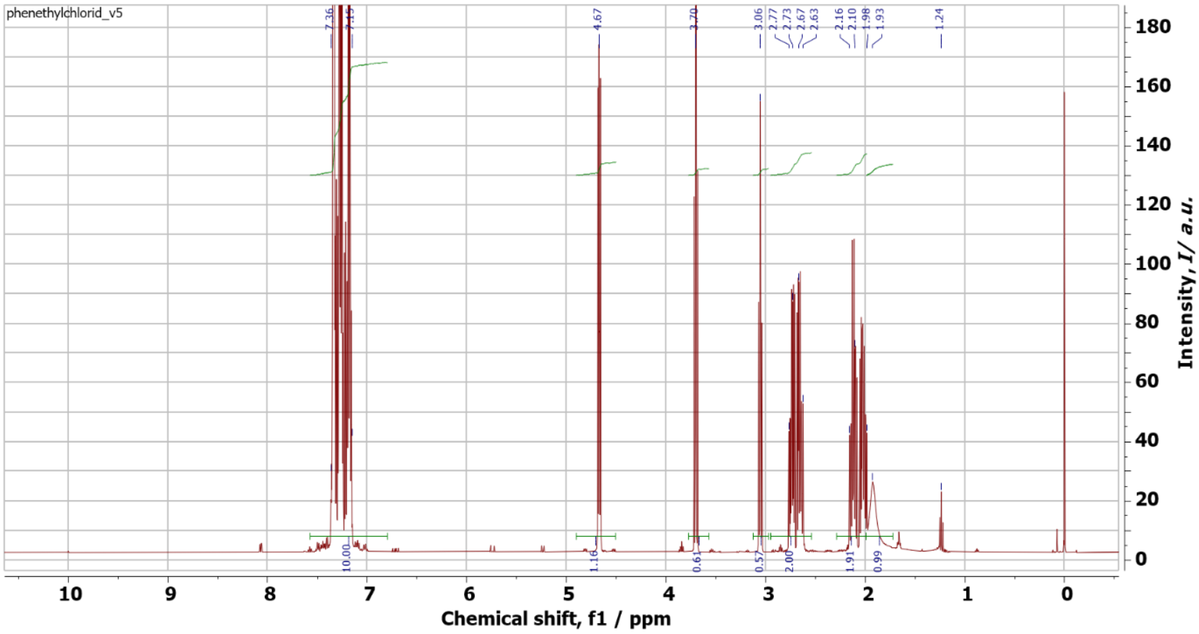

Figure 26: ^{1}H-NMR spectrum of crude reaction product of 1,3-Diphenylpropan-1-ol synthesis with feedback control acquired with 500 MHz NMR instrument.

3.7 Heat of reaction

A closer view to the relative concentrations from IHM plotted together with the internal reactor temperature basically reveals the redundancy of the NMR sampling for Grignard reactions with sufficient heat of reaction.

As shown in **Figure 27** the rise of temperature upon halide addition indicates the presence of reactive magnesium and thereby ongoing reaction. This suggests the applicability of a simple temperature sensor in case of exothermic reactions well below the boiling point of employed solvent and ideally under external cooling. However, this insight was mainly generated by using the feature-rich analysis method of NMR and also would miss occurring side-reactions, which can be assessed with IHM by increasing residues. Nonetheless, it could serve as a low-cost, broadly applicable method.

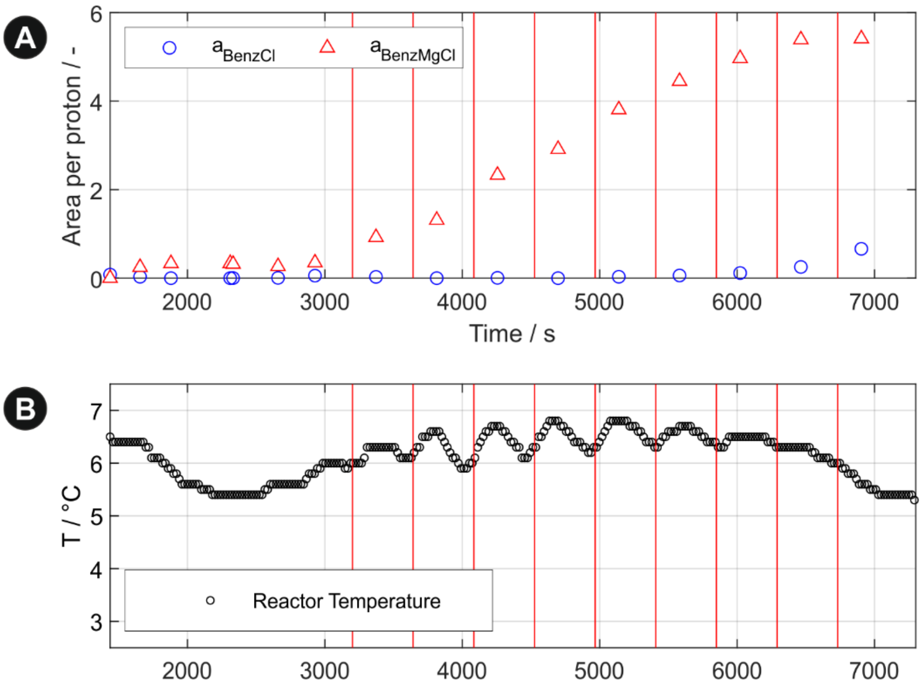

Figure 27: *Experimental data of 1,2-Diphenylethanol synthesis run showing: a) proton-normed species areas of benzyl chloride (BenzCl) and benzylmagnesium chloride (BenzMgCl) during Grignard formation including addition of BenzCl (red, vertical); b) Temperature inside the reactor in course of the reaction.*

References

[1] S. Steiner, J. Wolf, S. Glatzel, A. Andreou, J. M. Granda, G. Keenan, T. Hinkley, G. Aragon-Camarasa, P. J. Kitson, D. Angelone, L. Cronin, *Science* **2019**, *363*.

[2] D. Angelone, A. J. S. Hammer, S. Rohrbach, S. Krambeck, J. M. Granda, J. Wolf, S. Zalesskiy, G. Chisholm, L. Cronin, *Nat. Chem.* **2021**, *13*, 63-69.

[3] U. Tilstam, H. Weinmann, *Org. Process Res. Dev.* **2002**, *6*, 906-910.

[4] SDBSWeb : https://sdbs.db.aist.go.jp (National Institute of Advanced Industrial Science and Technology, 18.01.2021)

[5] M. R. Hollerbach, T. J. Barker, *Organometallics* **2018**, *37*, 1425-1427.

[6] C. E. Hartmann, V. Jurcik, O. Songis, C. S. Cazin, *Chem. Commun. (Cambridge, U.K.)* **2013**, *49*, 1005-1007.
